# Transient vascular and long-term alveolar deficits following a hyperoxic injury to neonatal mouse lung

**DOI:** 10.1186/1471-2466-14-59

**Published:** 2014-04-09

**Authors:** Alexandra B Firsova, Timothy J Cole, Richard Mollard

**Affiliations:** 1 Department of Biochemistry and Molecular Biology, Monash University, Clayton 3800, Victoria, Australia; 2 Current Address: Centre for Ideas and the Economy, Melbourne Business School, University of Melbourne, Parkville 3000, Victoria, Australia

**Keywords:** Hyperoxia, Lung development, Lung injury, Vascularization, Septation, Alveolarization

## Abstract

**Background:**

The lungs of very preterm human babies display deficits in alveolarization and vascularization as a result of the clinical use of high oxygen treatment (leading to hyperoxia) required to decrease the risk of mortality. Detailed analyses of the persistence of the respiratory deficits following this treatment and means to restore a normal state have not been investigated in full detail. In this study, high oxygen administration to neonatal mouse lungs was established as a proxy to hyperoxia in human preterm infant lungs, to better characterize the associated deficits and thus provide a means to assist in the development of treatments in the future.

**Methods:**

Ninety percent oxygen was administered to newborn mice for four consecutive days. The effects of this treatment upon alveolarization and vascularization were investigated by morphometric, histochemical, immunohistochemical and protein analyses at day five (D5), D28 and D56 postpartum.

**Results:**

Relative to control untreated lungs, septation of hyperoxic lungs was significantly reduced and airspaces were significantly enlarged at all stages examined. Furthermore, compared to controls, the number of secondary septa per tissue area was significantly reduced at D5, significantly increased at D28 and then the same as controls at D56. Analysis of vascularization parameters indicated a reduction in mature blood vessel number and the amount of Pecam1 at D5. Both of these parameters returned to control levels by D28.

**Conclusions:**

This study suggests that administration of high oxygen to underdeveloped lungs has a transient reductive effect on secondary septal number and pulmonary vascularization and a significant long-term reduction in alveolarization persisting into adulthood. This model can be used for future research of premature lung disease therapies in humans, addressing these short term septal and vascular and long term alveolar deficits, specifically relating to injury by hyperoxia.

## Background

Preterm birth occurs in approximately 10% of human pregnancies between 20 and 37 weeks of gestation. During this period, human lung development proceeds through the canalicular and saccular stages and then on to the alveolar stage. This process is essential for gas-exchange surface formation and optimization, and involves epithelial wall thinning, epithelial cell differentiation and lung vascularization [1-5]. Because the human lung continues to develop throughout the late stages of gestation, very preterm babies are born with underdeveloped lungs. This underdevelopment manifests a structural immaturity that does not permit efficient gas exchange, and hence reduces bloodstream oxygen levels. Although the cellular and molecular processes within the saccular and alveolar stages of mice and humans show commonality, the developmental timing of these stages is different between species. The saccular stage of mouse lung development commences at approximately embryonic day (E)17.5 and is not completed until approximately postnatal day five, whereas in humans, the saccular stage commences during the 24th week of gestation and is completed by birth [4,6]. The mouse alveolar stage starts at five days and is completed at approximately three weeks postpartum, whereas human alveolarization commences at 36 weeks of gestation and ends after a few postnatal years [4,6,7]. This developmental anachrony between mice and humans means that newborn mouse lungs may be conveniently used as a model system for studying disease processes associated with preterm birth human saccular stage lungs.

Often in cases of human preterm birth, the gas exchange tissues are underdeveloped and in order to provide an adequate amount of oxygen in the bloodstream, oxygen concentration in the respired air must be increased, leading to bronchopulmonary dysplasia (BPD). The influence of this high oxygen exposure shortly after birth on lung structure, as well as on proteins involved in vascularization and alveolarization, has been investigated in a number of studies, using experimental animal models [8-12]. Generally it is understood, that hyperoxia affects various parameters of alveolarization, with the following reductions observed: mean linear intercept, mean alveolar surface, secondary crest number and alveolar number [8,10,12-14]. A significant reduction in vascularization after hyperoxia in newborn animals has also been reported. More specifically, treatment with 80% oxygen reduces the number of pulmonary blood vessels, as well as *VefgA* mRNA and VEGF protein levels [6,8,11]. Lung fibrosis and elastic and collagen fiber disorganization have also been observed after hyperoxia exposure [12,13,15]. Previous studies have demonstrated that changes in elastin depositions affect alveolar formation [16]. Although it can be concluded that most essential tissues involved in gas exchange are affected by hyperoxia shortly after exposure, the precise timing and duration of changes consequential to hyperoxia remain undetermined. That is, whether these alterations to lung structure, cellular and molecular parameters of the respiratory tissues are temporary, and whether any of them persist into the adulthood, remains to be fully investigated.

The aim of this study, therefore, was to characterize in detail temporal changes in saccular stage alveolar and vascular development associated with hyperoxia in mice. Newborn mice have been used as a model for human pre-alveolarization state of lung development, when humans are most likely to develop BPD when treated with high oxygen. Mice were exposed to 90% oxygen from E17.5 to D4 and analyses of alveolar, vascular and molecular parameters were performed at three time points: day five (D5), D28 and D56 postpartum. Both permanent and transient alterations to alveolar and vascular architecture and molecular biology were recorded during this period.

## Methods

### Mouse model of hyperoxia

All animal experimental methodology was approved by the School of Biomedical Sciences Animal Ethics Committee at Monash University, Australia. Pregnant C57Bl/6 J females housed in specific pathogen free (SPF) conditions were brought into SPF procedure rooms of the animal house at E14 and placed into clear plastic chambers (length × width × height = 425 × 266 × 185 mm; floor area 800 cm^2^ [Techniplast]). E0.5 was taken to represent the morning of vaginal plug identification. Pregnant females were placed in the high oxygen environment at E17.5 so that: (i) the pups would be born directly into the high oxygen environment and (ii) the mothers would have time to adjust to the high oxygen environment and thus reduce stress prior to parturition. Oxygen concentrations were recorded using a portable gas analyzer (Servomex 5200 Multi-Purpose) at 15 minute (min) intervals throughout treatment. Oxygen levels were maintained at 90 ± 5%, and returned to 20% (i.e. room control concentrations) for 30 min each day during compulsory animal monitoring periods and rotating of natural mothers and foster mothers. The natural mothers and foster mothers only, not the pups, were rotated between hyperoxic and normoxic conditions to reduce stress and toxicity associated with hyperoxia to them. The mothers were not the subject of this study, only their pups. Pups and mothers were housed at one litter per chamber for 24 hours (h). The number of pups per chamber was equalized between hyperoxia and normoxia (control) groups. Oxygen flow was turned off gradually (reducing final oxygen concentration by 10% each 30 min) on the afternoon of postnatal day 4. Pups were left to recover in room air for one day before the first set of samples was collected. Pups remained in the same cages until weaning at three weeks of age. Pups were weighed once a week from D7 to D56. Both males and females were culled by cervical dislocation for analysis on postnatal days five, 28 and 56. Lungs were isolated at each time point for analysis.

### Lung morphometric analysis

Lungs were pressure-fixed *in situ* via instillation with 4% paraformaldehyde (PFA) in phosphate buffer saline (PBS) at 20 cm (H_2_O) via a cannula inserted into the trachea [17]. PFA-saturated lungs were then excised with rib-cages and post-fixed for 24 h in 4% PFA at 4°C. The following day, lungs were removed and placed into Zamboni’s fixative for 24 h at room temperature (RT) [18]. Lungs were embedded in paraffin and serially sectioned as 5–6 μm serial coronal sections in the ventral to dorsal plane. Slides were randomly picked from each group for staining and analysis. Sections on glass slides were deparaffinized by immersion in xylene (3 × 5 min), 100% ethanol (3 × 5 min) and tap water (1 min). Lung sections were stained for histological analyses using four methods: hematoxylin and eosin stain (Mayer’s hematoxylin – 3 min, acid alcohol – 3 seconds (s), Scott’s tap water – 10 s, eosin – 3 min, rinsing in tap water between solutions), Masson’s trichrome stain (Celestin blue – 5 min, Mayer’s hematoxylin – 3 min, acid alcohol – 3 s, Biebrich scarlet/acid fuchsin – 5 min, 1% phosphomolybdic acid – 5 min, aniline blue – 5 min, rinsing in tap water between solutions), periodic acid and Schiff’s (PAS) stain (periodic acid – 5 min, Schiff’s reagent – 5 min, hematoxylin – 5 min, acid alcohol – 3 s, Scott’s tap water – 10 s, rinsing in tap water between solutions) and modified Weighert’s elastin stain (0.25% potassium permanganate – 5 min, 5% oxalic acid – 3 s, resorcin-fuchsin – overnight, tartazine in saturated picric acid – 3 min, rinsing in tap water between solutions) [18,19]. All stains were prepared according to standard protocols [19]. After staining, slides were dehydrated in 100% ethanol and xylene (3 × 5 min) and mounted in DPX mounting medium (BDH Laboratory Supplies, #360294H) [19]. Five to seven photographs were taken per section (depending on lung size) using a light microscope (Olympus CKX41) and digital camera to assess the entire alveolar area [15]. Photographs of lung sections were analyzed for the following characteristics: mean linear intercept (L_m_), tissue area, alveolar number, elastin-positive blood vessel number per tissue area, secondary septum (crest) number per tissue area, elastin-positive tissue per total tissue area, the presence of mucus and mucin-secreting cells, the presence of collagen depositions and fibrosis. To evaluate the number of alveoli, three vertical and two horizontal lines were drawn randomly across the lung section image. The total lengths of vertical and horizontal lines were equal. The summary length of all lines per image was 3.63 mm. The average number of alveoli was calculated from the number of airspaces (including alveolar ducts, but excluding airways) appearing on those lines [15]. Mean linear intercept (L_m_) was calculated from measurements of the lengths of airspaces (both alveoli and alveolar ducts, but excluding airways) falling on top of these lines. The number of vertical measurements was equal to the number of horizontal measurements. Secondary septa and blood vessels were detected by the presence of elastin that was stained black by resorcin-fuchsin (Weighert’s elastin stain) [20,21]. The number of secondary septa and the amount of elastin were calculated in relation to the area of tissue per total area of image. The number of blood vessels was calculated both per total image area and per tissue area. The tissue area was calculated using Image-Pro Plus software (Media Cybernetics) by selecting all colored pixels on the section image and then transferring them into microns according to the scale bar. The amount of elastin was calculated by determining the total percentage of elastin-positive tissue using Image-Pro Plus.

### Immunohistochemistry

Lung paraffin sections were analyzed for the immunoreactivity to Pecam1 and Vegfa. Lung sections were deparaffinized and rehydrated as described above, boiled in 0.01 M sodium citrate solution for 20 min, left to cool at RT for 30 min and then rinsed in distilled water (dH_2_O). Slides were then incubated in 3% H_2_O_2_ for 10 min, rinsed in dH_2_O and blocked with 5% goat serum at 4°C overnight. Lung sections were incubated with primary and secondary antibodies for one hour (1 h) at RT in a wet chamber. Primary antibodies were used at the following concentrations: Pecam1 (Abcam, #ab28364) at 1:100, Vegfa (Santa Cruz, #sc-152) at 1:750. Secondary goat anti-rabbit biotinilated IgG antibody was used at 1:1000 (Invitrogen, #B2770). Streptavidin-HRP (DAKO, #P0397) complex solution was applied and incubated for 30 min at RT. Sections were washed with PBS containing 1% Tween-20 (PBST) for 15 min (3 × 5 min) after each incubation. DAB-chromogen in 1 ml of DAB-substrate buffer complex (DAKO, #K3468) was applied for 5 min for Pecam1 antibody and for 5–15 min for Vegfa antibody. Sections were counter-stained with Mayer’s hematoxylin, dehydrated with ethanol and xylene, and mounted in DPX mounting medium.

### Western blot analysis

Lung samples were snap-frozen in dry ice and stored at -80°C for a maximum of 12 months. Frozen samples were thawed and homogenized in 1 ml of radioimmunoprecipitation assay buffer for 30 min, and centrifuged at 14000 rpm for 20 min at 4°C. Protein concentration was measured in the supernatant using the Bradford protein assay. Samples (the supernatant) were diluted in dH_2_O to equalize protein concentrations, and further diluted 1:1 in reducing sample buffer and boiled for 5 min at 100°C, and then either stored at -20°C, or loaded into 7% or 12% polyacrylamide gels for Western blot analysis as previously described [22]. Primary rabbit anti-mouse antibodies were used at the following concentrations: Pecam1 (Abcam, #ab28364) at 1:100, Vegfa (Santa Cruz, #sc-152) at 1:200, or β-actin (Actb, Sigma, #A-2066) at 1:500. Membranes were manually exposed to X-ray film (Fujifilm); the films were developed and scanned (Canon Image Scanner), and the bands were analyzed using ImageQuant TL gel analysis software.

### Statistical analysis

A univariate general linear model, where group (i.e. hyperoxia/normoxia at each time point) was allocated as the main factor and animal – as the random factor (five to seven measurements per animal were taken, or in the case of mean linear intercept – 160 measurements per animal), was applied for analysis of variance to determine significant differences in morphometric characteristics between hyperoxic and control lungs at different stages using the SPSS statistical package (PASW, IBM). The “litter” was not included as a random variation factor since only one to two mice per litter were used at each time point. The interaction analysis was performed, where group and time were the main effects. Data plots were drawn in Microsoft Excel. Error bars indicate standard error of the mean (SEM), and were calculated from average values per mouse without taking into account variations within each animal. One-way analysis of variance (ANOVA) was used to determine significant differences in mouse body weight, protein levels and the number of examined cell types between hyperoxic and control lungs at different stages.

## Results

### Sample collection distribution

Twenty-seven neonatal pups were exposed to 90% oxygen for four days and 28 pups were maintained as controls (normoxic mice). A total of 19 mice (nine males and ten females) were culled at five days of age with 12 lungs from six males and six females pressure-fixed for morphometric analysis and seven lungs from three males and four females frozen for protein analysis. Fifteen mice (nine males and six females) were culled at 28 days of age (eight lungs from five males and three females for morphometric analysis and seven lungs from four males and three females for protein analysis). Twenty-one mice (11 males and ten females) were culled at 56 days of age (13 lungs from six males and seven females for morphometric and eight lungs from five males and three females for protein analysis). All 55 mice used in this data set were treated and analyzed over a period of ten months in three independent experimental pools.

### Animal wellbeing

**Figure 1:**
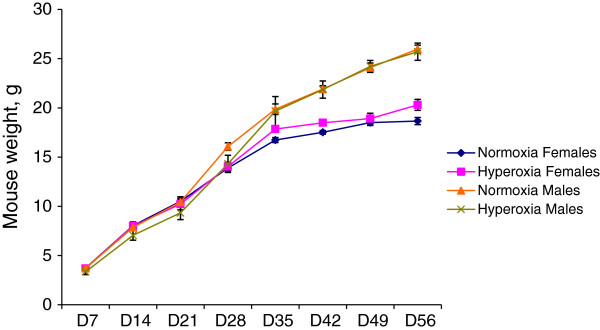
**Body weight of male and female mice with (hyperoxia) or without (normoxia) high oxygen treatment.** Error bars represent SEM, from D7 to D28 n = 6; from D35 to D56 n = 4.

During the eight weeks following treatment both normoxic and hyperoxic mice gained weight similarly (Figure 1) and did not demonstrate any signs of distress (data not shown). An exception was that during the first two postnatal weeks, all hyperoxic mice demonstrated an abnormal breathing pattern when placed under stress, e.g. when the bedding was being changed or when mice were being weighed, breathing heavily with a ‘clicking’ noise. This sound was not heard from normoxic mice during the same stress conditions. There was no significant difference in mouse body weight between normoxic and hyperoxic mice throughout the whole experiment (Figure 1). Males and females were compared separately, as males gained weight faster than females after 28 days.

### Morphometric analysis of lungs after hyperoxia

#### *Gender comparison*

Males and females were first compared separately for different morphometric parameters, however, no significant gender differences were observed in parameter variations (data not shown). Therefore, for further analyses males and females were pooled in both the hyperoxia and normoxia groups.

#### *Persistent changes in alveolarization*

**Figure 2:**
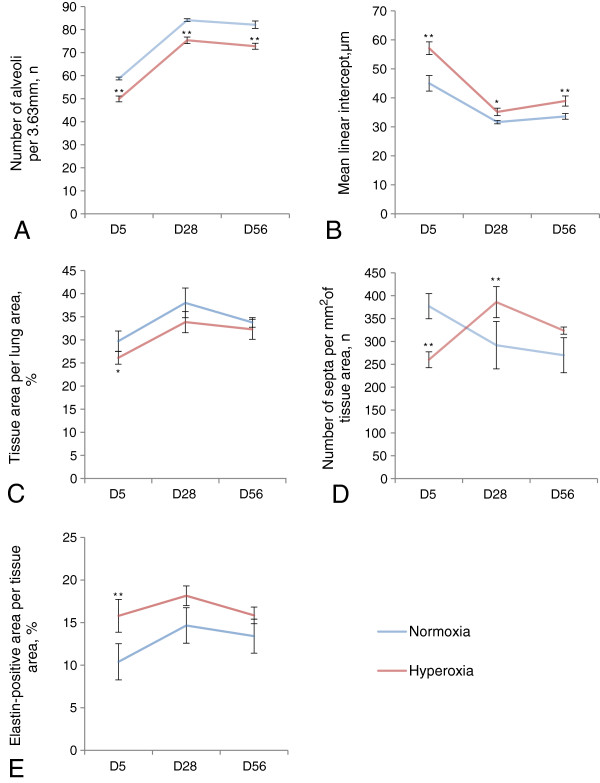
**Statistical data for morphometric analysis of lungs after hyperoxia.** Control (normoxia) – blue line, hyperoxia – red line. **A**. Average number of airspaces falling on a 3.63 mm line drawn across the lung image, representing the average number of alveoli. **B**. Mean linear intercept, representing the average size of alveoli and alveolar ducts in 1D. **C**. Tissue area per lung area, representing the average amount of tissue on the area of lung section image. **D**. Average number of secondary septa per 1 mm^2^ of lung tissue area on the lung section image. **E**. Average ratio of the area stained positively for elastin to lung tissue area. The differences between normoxia and hyperoxia values were calculated using a general linear model (SPSS). P < 0.05 is marked as one asterisk, and P < 0.01 is marked as two asterisks. Error bars indicate SEM, n = 4-6. Plot interactions are described in the text.

Persistent differences in specific lung morphometric features were observed in respect to normoxia versus hyperoxia. Hyperoxia induced a significant decrease in average alveolar number, measured as airspaces per linear 3.63 mm, and this decrease was observed at each time point examined: postpartum day five (D5), 49.88 ± 1.25 (hyperoxia, n = 6) versus 58.75 ± 0.63 (normoxia, n = 6), P < 0.01; D28, 75.35 ± 1.42 (hyperoxia, n = 4) versus 84.10 ± 0.61 (normoxia, n = 4), P < 0.01; and D56, 72.52 ± 1.30 (hyperoxia, n = 6) versus 82.35 ± 1.61 (normoxia, n = 6), P < 0.05; (five to seven measurements per animal; Figure 2A). Accordingly to this alveolarization decrease, hyperoxia induced a significant increase in mean linear intercept (L_m_) at each time point examined: D5, 57.13 ± 2.20 μm (hyperoxia, n = 6) versus 45.02 ± 2.68 μm (normoxia, n = 6), P < 0.01; D28, 35.15 ± 1.30 μm (hyperoxia, n = 4) versus 31.64 ± 0.58 μm (normoxia, n = 4), P < 0.01; and D56, 38.92 ± 1.75 μm (hyperoxia, n = 6) versus 33.61 ± 0.98 μm (normoxia, n = 6), P < 0.01 (160 measurements per animal; Figure 2B). These measurements of alveolar number and L_m_, therefore, demonstrate that hyperoxia in mouse pups causes an immediate deficit in septation and that this decrease is not reversed with time.

#### *Temporary changes in tissue area, secondary septa and elastin amount*

**Figure 3:**
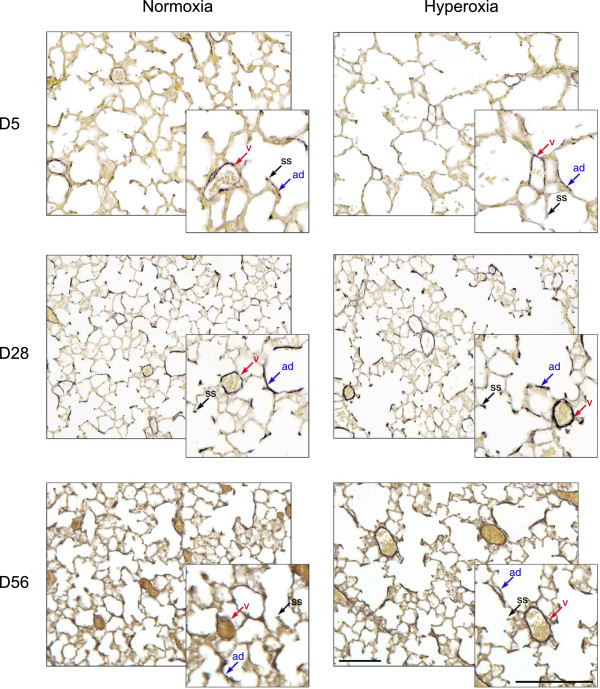
**Weighert’s elastin staining of control (normoxia) and hyperoxic (hyperoxia) lungs at D5, D28 and D56.** Elastin is stained black. Cytoplasm and nuclei of all cell types are stained yellow. Secondary septa (ss) are marked with black arrows, blood vessels surrounded by a layer of elastin (v) are marked with red arrows, and elastin depositions in the alveolar walls (ad) are marked with blue arrows. Scale bar = 100 μm.

In contrast to persistent changes in alveolar number and L_m_, other parameters measuring postnatal lung development demonstrated transient and significant changes that returned to control levels with time. For example, lung tissue area per image in hyperoxic mice was significantly decreased compared to normoxic mice at D5, but was not significantly different at D28 and D56: D5, 26.12 ± 1.39% (hyperoxia, n = 6) versus 29.71 ± 2.21% (normoxia, n = 6), P < 0.05; D28, 33.84 ± 2.28% (hyperoxia, n = 4) versus 38.01 ± 3.21% (normoxia, n = 4), P = 0.05; and D56, 32.28 ± 2.17% (hyperoxia, n = 6) versus 33.77 ± 1.04% (normoxia, n = 6), P > 0.05; (Figure 2C). The number of secondary septa per tissue area in hyperoxic mice was significantly reduced compared to normoxic mice at D5, significantly increased at D28, but was not significantly different at D56 (measurements are given as septum number per mm^2^ of tissue): D5, 259.94 ± 17.36 (hyperoxia, n = 6) versus 377.12 ± 27.57 (normoxia, n = 6), P < 0.01; D28, 385.98 ± 33.92 (hyperoxia, n = 4) versus 291.83 ± 51.75 (normoxia, n = 4), P < 0.01; and D56, 323.74 ± 7.88 (hyperoxia, n = 6) vs 269.73 ± 38.32 (normoxia, n = 6), P ≥ 0.05; (Figure 2D). Finally, elastin deposits were measured as a total elastin amount to provide information regarding changes in alveolar walls, septa and blood vessels, and whether these changes were persistent. These deposits on the tips of secondary septa, around blood vessels and within alveolar walls (Figure 3) were measured by Image-Pro Plus as a total elastin-positive area per lung tissue area counted on a lung section area of 0.564 mm^2^ and were found to be significantly increased in hyperoxic lungs compared to normoxic lungs at D5; however, no significant differences were observed at D28 and D56: D5, 15.80 ± 1.92% (hyperoxia, n = 6) versus 10.40 ± 2.12% (normoxia, n = 6), P < 0.01 (Figure 2E). These results indicate that processes affecting lung tissue area, secondary septa formation per tissue area and elastin deposition during postnatal lung development, are significantly affected during high oxygen treatment, but subsequent compensatory mechanisms return these parameters to control levels.

#### *Timeline analysis of alveolarization parameters*

Timeline analysis was performed to examine more specifically how morphometric parameters affected during high oxygen treatment were maintained or altered with time compared to normal development and growth. To do this, changes in normoxic mouse development from D5 to D28 to D56 were analyzed and then compared to those of hyperoxic mice using interaction analysis. With respect to the number of alveoli, there was no significant interaction between normoxia and hyperoxia groups, with the number of alveoli increasing from D5 to D28 and then showing no change from D28 to D56 (Figure 2A). Mean linear intercept (L_m_) decreased from D5 to D28 in both normoxic and hyperoxic mice (P < 0.05) and then increased from D28 to D56 (P < 0.05), with no significant interaction between the plots (Figure 2B). There was also no significant interaction between the plots for the lung tissue area, i.e. time had similar effects on the lung tissue area in both normoxic and hyperoxic groups (Figure 2C). Although lung tissue area of both normoxic and hyperoxic mice both increased from D5 to D28 (P < 0.05), it did not change from D28 to 56 in the normoxic and hyperoxic mice (Figure 2C). The plots for the number of secondary septa per tissue area and the amount of elastin per tissue area had a significant interaction (P < 0.05). The number of secondary septa per tissue area decreased from D5 to D28 in normoxic mice (P < 0.05), but increased during this time in hyperoxic mice (P < 0.05; Figure 2D). Between D28 and D56 the number of secondary septa decreased in hyperoxic mice (P < 0.05) and stayed at the same level in normoxic mice. Lastly, the amount of elastin per tissue area increased from D5 to D28 in normoxic mice (P < 0.05), but not in hyperoxic mice, where it was significantly higher at D5 and persistently remained high, with a significant interaction between the plots (P < 0.05, Figure 2E). These data demonstrate that some morphometric parameters (number of alveoli, L_m_ and lung tissue area) develop similarly in hyperoxia and normoxia groups during the time examined, while other parameters (amount of elastin and the number of secondary septa) do not.

### Analysis of the lung vascularization after hyperoxia

#### *Blood vessel number*

**Figure 4:**
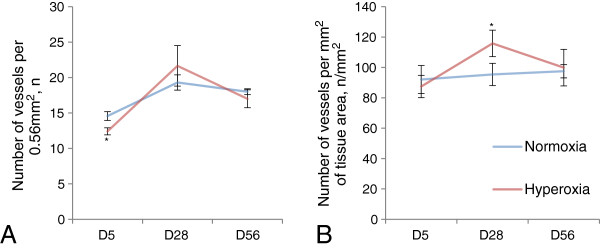
**Statistical data for vascularization analysis of lungs after hyperoxia.** Control (normoxic lungs) – blue line, hyperoxic lungs – red line. **A**. Average number of elastin-positive blood vessels on the lung section image. **B**. Average number of elastin-positive blood vessels per lung tissue area. The differences between normoxia and hyperoxia values were calculated using a general linear model (SPSS). P < 0.05 is indicated as one asterisk. Error bars indicate SEM, n = 4-6. Plot interactions are described in the text.

In addition to studying morphometric changes of the lung parenchyma following high oxygen treatment, changes to the accompanying lung vasculature were also examined. Significant differences were observed with respect to blood vessel number per lung section area (i.e. including airspaces) and blood vessel number per total tissue area (i.e. excluding airspaces), and these differences were not dependent on mouse gender. For example, the number of blood vessels surrounded by an observable elastin layer (Figure 3) per 0.564 mm^2^ of lung section area was significantly decreased at D5 yet no differences were observed at D28 or D56: D5, 12.41 ± 0.50 (hyperoxia, n = 6) versus 14.6 ± 0.62 (normoxia, n = 6); P < 0.05; (Figure 4A). With respect to blood vessel number per total tissue area, there was no significant difference at D5 and D56, yet at D28 hyperoxic lung vessel numbers per total tissue area were significantly increased compared to normoxic lungs: D28, 115.81 ± 8.70 (hyperoxia, n = 4) versus 95.38 ± 7.34 (normoxia, n = 4); P < 0.05 (Figure 4B).

#### *Timeline analysis for blood vessel number*

Timeline analysis was similarly performed as above to examine more specifically how blood vessel parameters affected during high oxygen treatment were maintained or altered with time compared to normal developmental events. With respect to the number of blood vessels per lung section area (including airspaces), there was a significant increase in blood vessel number in both normoxia and hyperoxia groups from D5 to D28 (P < 0.05 for both groups). Subsequently, from D28 to D56, there was a significant reduction in blood vessel number in the hyperoxia group (P < 0.05), yet no reduction in the normoxia group. Despite these differences between D28 to D56, and according to interaction analyses, charts were parallel, i.e. time had a similar effect on the number of blood vessels in both treatments (Figure 4A). With respect to the number of blood vessels per lung tissue area, no significant differences were seen in the normoxia group from D5 to D56. However, the hyperoxia group demonstrated a significant increase from D5 to D28 (P < 0.05) and a significant decrease from D28 to D56 (P < 0.05; Figure 4B). There was a significant interaction between normoxia and hyperoxia charts (P < 0.05). Together, these data indicate that early hyperoxia treatment exerts either a direct or indirect latent stimulatory effect upon vascular development (observable at D28) and that these effects are subsequently interpreted and normalized with respect to surrounding tissue area by D56.

#### *Protein analysis for Pecam1 and Vegfa*

**Figure 5:**
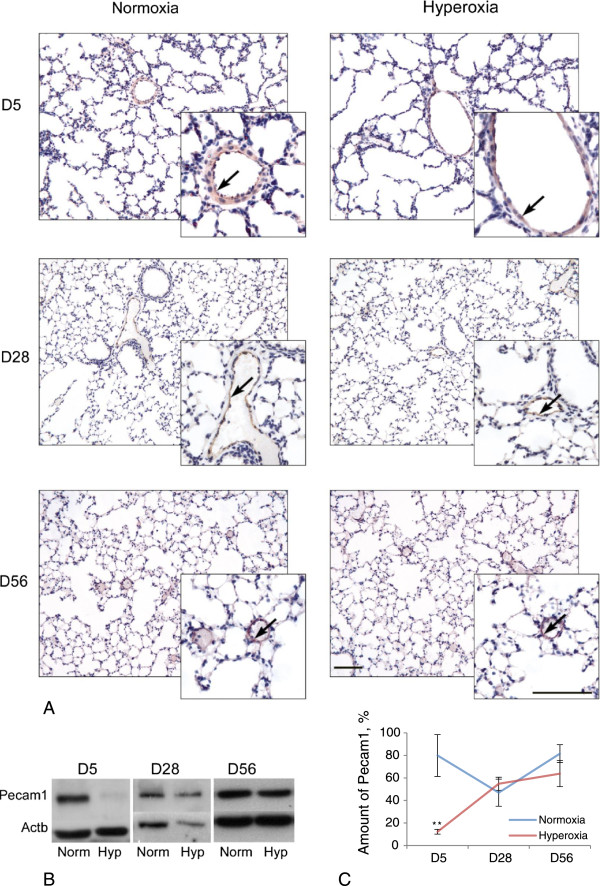
**Pecam1 protein analysis. A**. Immunohistochemistry for Pecam1. Brown staining indicates localization of Pecam1 in the lung of normoxic and hyperoxic mice at three time points (D5, D28 and D56). Mayer’s hematoxylin staining (dark blue) indicates nuclei. Blood vessels are marked with arrows. Scale bar = 100 μm. **B**. Western blot for Pecam1 (140 kDa) in the lung at three time points (D5, D28 and D56). Actb is used as the loading control (42 kDa). Norm – Normoxia, Hyp – Hyperoxia. **C**. Levels of Pecam1 per total protein (relative to Actb) according to the Western blot. Error bars represent SEM, n = 3-4. Significant difference is marked with two asterisks (P < 0.01, n = 3-4).

**Figure 6:**
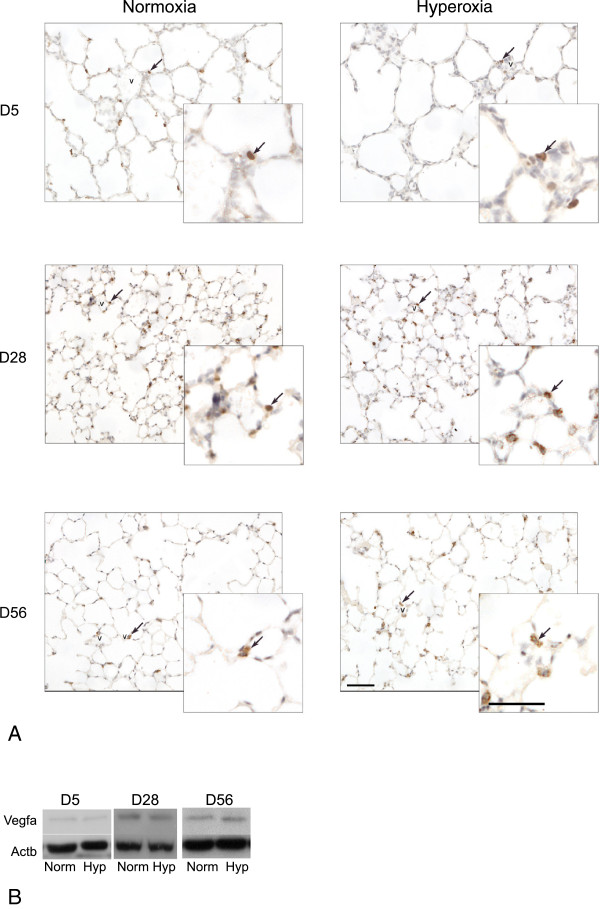
**Vegfa protein analysis. A**. Immunohistochemistry for Vegfa on lung sections of normoxic and hyperoxic mice at three time points (D5, D28, and D56). Brown staining indicates Vegfa (marked with arrows) and blue staining (Mayer’s hematoxylin counterstain) indicates nuclei. Vegfa-positive cells associated with blood vessels are marked with arrows. Blood vessels are marked with “v”. Scale bar = 50 μm. **B**. Western blot for Vegfa (21 kDa). Actb is used as the loading control (42 kDa). Norm – Normoxia, Hyp – Hyperoxia.

With respect to mechanisms affecting changes to vascular development: (i) Pecam1 immunoreactivity as an indicator of endothelial differentiation status and as a blood vessel specific marker, and (ii) Vegfa immunoreactivity as an indicator of the level of influence of alveolar epithelial type II cells (AECII) on vascular development, were investigated by both Western blot analysis and immunohistochemistry. Pecam1 was localized predominantly in blood vessel walls (i.e., endothelial cells, Figure 5A). Pecam1 protein levels standardized to the loading control β-actin were significantly lower in hyperoxic lungs compared to normoxic lungs at D5, while no significant differences were observed at D28 and D56: D5, 12.22 ± 2.09% (hyperoxia, n = 3) versus 79.92 ± 18.57% (normoxia, n = 4); P < 0.01 (Figure 5B, C). Vegfa was localized predominantly in lung epithelial cells (Figure 6A), which is consistent with the expression of Vegfa by AECII [23]. Some Vegfa-immunoreactive epithelial cells were associated with blood vessels (Figure 6A). No significant differences in Vegfa protein levels were observed at any time point examined (Figure 6B).

### Assessment of respiratory fibrosis and airway mucus following hyperoxia

**Figure 7:**
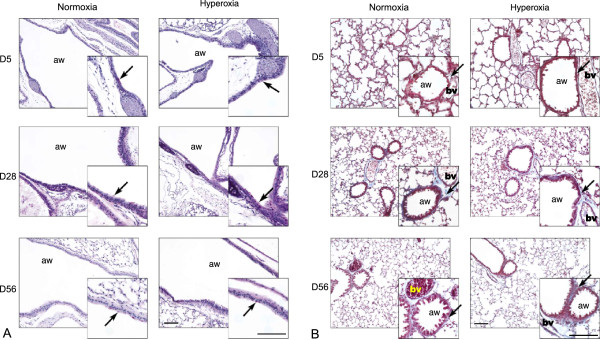
**Assessment of airway mucus and respiratory fibrosis following hyperoxia. A**. Representative images of periodic acid-Schiff’s reagent staining of normoxic and hyperoxic lung airways, counterstained with Mayer’s hematoxylin (blue). Mucin-secreting cells are stained purple and are marked with arrows. No mucus deposits are present in the airways (aw) of either normoxic or hyperoxic mice. Scale bar = 100 μm. **B**. Representative images of Masson’s trichrome staining of normoxic and hyperoxic lungs. A collagen layer (stained blue) around the airways (aw) and blood vessels (bv) is present in both normoxic and hyperoxic lungs and is marked with black arrows. Nuclei are stained purple, cytoplasm is stained red. No abnormal collagen deposits (i.e. thickening of lung tissue area) are observed in hyperoxic lungs at any stage. Scale bar = 100 μm.

As described above and following hyperoxia, an increase in lung tissue elastin staining was observed at D5, but levels returned to control values by D28 (Figures 2 and 3). Further, morphological analyses of lung tissue showed no overt visible respiratory damage or pathological changes in the lung after hyperoxia. No airway mucus and no obvious fibrosis or abnormal collagen deposits were observed at any time point examined (Figure 7). Mucus-secreting cells were observable in the trachea (Figure 7A), and collagen deposits were localized around blood vessels (Figure 7B), which indicated healthy lung morphology.

## Discussion

In this study we show that both alveolarization and vascularization of neonatal mice are affected by hyperoxia, but to different degrees and to some extent independently to each other. Septation parameters (number of alveoli and L_m_) were persistently affected by hyperoxia, whereas vascularization was only affected temporarily. A temporarily reduced rate of secondary septal number was associated with a reduction in the number of alveoli and an increase in L_m_, whereas temporarily reduced levels of vascular marker Pecam1 were associated with a reduction in the total number of blood vessels per section area. The reduced blood vessel number per tissue area observed at D5 had normalized by D28 and this measure was associated with a rise in Pecam1 levels. The return of some parameters to control levels could be attributed to a shorter hyperoxia exposure period here compared to other studies: four days here and up to 10 days in other studies [8,10,13,14].

Neonatal mice treated with high oxygen just prior to birth and for four days displayed no signs of pain or distress, and did not lose weight in comparison to normoxic control mice. No difference was also observed in behavioral patterns (movement in the cage), and there was no incidence of hyperoxia-induced mortality. After high oxygen treatment heavy and loud breathing was noticed in pups during handling, however, no mucus was observed in the airways after mucin staining of lung sections. It was, therefore, concluded that the reason for noisy breathing was not due to lower airway obstruction. Previous studies have shown that the method of paraformaldehyde lung infusion and PAS staining is effective for observing lung mucus [24]. There were also no signs of fibrosis observed in the lung after collagen staining of lung sections. Although not determined here, whether the airway noises were associated with changes to upper airway structures, for example, larynx, pharynx or nasal structures, merits future analysis. Previous studies have demonstrated an increase in fibrosis in the lungs after hyperoxia, as well as disorganization of collagen deposits [12,13]. The absence of these changes in the current study could be explained by an initial very short oxygen exposure *in utero* as well as a shorter treatment (four days compared to ten days).

Pups were exposed to hyperoxia just prior to birth and for the first four days postpartum, corresponding to the saccular stage of lung development [6]. Alveolarization in mice commences at approximately D5. During the alveolar stage, secondary septa begin to form and reshape the alveolar wall. Hyperoxia exposure in this study, therefore, directly affects processes associated with the saccular stage of lung development and this exposure has ramifications that were then measured in the alveolar stage. A lower alveoli number and higher L_m_ were observed during normal development at D5, compared to D28 and D56. This may be explained by the fact that between D5 and D28 the main steps of alveolarization occur: the number of secondary septa per tissue area reduces as the number of alveoli increases and L_m_ reduces. Between D28 and D56 secondary septation overtly ceases, therefore, and as observed here L_m_ and the number of alveoli do not markedly change. At D5, the number of secondary septa per tissue area in hyperoxic mice was reduced compared to control levels, as was the number of alveoli, and this was accompanied by a higher L_m_. During the process of adaptation to normal oxygen conditions, the rate of secondary septation then increased, such that the same number of secondary septa per tissue area as normoxic mice at D5 was reached by D28. However, L_m_ remained high and alveolar number did not reach control levels even by D56. This means, that there was a reduced contact area between air and lung tissue at all tested time points, including D56, which would make breathing less efficient. Therefore hyperoxia here caused a chronic lung septation and respiratory deficit: the deficit persisting into adulthood. These results confirm previous studies of the effects of hyperoxia on alveolarizaton [8,10,12-14].

No significant difference between tissue area in normoxic and hyperoxic mice at D28 and D56 was observed, indicating a partial recovery of the lung tissue. The tissue area values however affected other parameters of lung morphometry that were measured in relation to these values, such as the amount of elastin, and secondary septa and blood vessel number. Less elastin per tissue area was observed at D5 in normoxic mice compared to hyperoxic mice. However elastin levels increased by D28, whereas in hyperoxic mice they were high at each time point examined. It can therefore be concluded that hyperoxia increased elastin levels in the neonatal lung. This confirms earlier studies that demonstrated that elastin deposits in the lungs increase after both ventilation and hyperoxia [25,26]. Elastin is produced by vascular smooth muscle cells and surrounds mainly arteries, whereas veins have less elastin, and in small capillaries there is very little or no elastin [27]. Elastin is also present on the tips of secondary septa [4]. Because the total number of blood vessels per tissue area was not altered and secondary septal number per tissue area was decreased at D5, the relative increase in elastin levels in the D5 hyperoxic mice, therefore, appears to be attributable to higher deposits at pre-existing sites and also within alveolar walls. The disorganized deposition of elastin and increased interstitial elastin fibers have been reported in previous studies, while changes in the total amount of elastin were not [12,15]. The difference in our findings could be attributed to the timing of hyperoxia exposure (E17.5-D4 vs D5-13 and D0-D11), and/or species-specific difference [12,15].

Since the tissue area was reduced in hyperoxic mice at D5, the number of elastin positive blood vessels was therefore evaluated not only directly, but also in relation to tissue area. As the tissue area increased to control levels at D28 in hyperoxic mice, the number of elastin-positive blood vessels also increased to reach control levels. The lack of elastin-positive vessels after hyperoxia at D5 may be related to reduced tissue area and reduced Pecam1 levels, which were also observed at this stage. It must be noted, however, that counting only elastin-positive vessels does not include all types of blood vessels and that the reduction in elastin positive blood vessels may indicate the presence of smaller or less mature blood vessels. Blood vessel growth was hyperstimulated in the hyperoxic mouse lung after D5 such that by D28 the earlier deficit had disappeared and both the number of blood vessels per section area and the Pecam1 levels had reached control levels. The amount of Vegfa remained unchanged in hyperoxic lungs relative to normoxic lungs at all time points examined. A significant reduction in Vegfa levels has been demonstrated in previous studies after ten days of high oxygen treatment (80%), compared to four days of high oxygen treatment (90%) with a brief recovery period in this study [8]. The differences in findings between previous studies and the study here may be attributable to the differences in duration and timing of hyperoxia exposure. By D56 there was no significant difference observed in any vascular parameters measured. Therefore these data indicate that the mechanisms regulating septation and vascularization in the mouse lung display a certain degree of independence.

## Conclusion

In conclusion, the hyperoxia model of four days of 90% oxygen in newborn mice caused deficits in septation, lung tissue area, alveolarization and respiratory vascularization. While the deficits in secondary septa, lung tissue area and vascularization were temporary, the deficit in alveolarization was permanent. Therefore, despite the absence of (i) weight loss, (ii) mucus in the airways, and (iii) fibrosis, four days of 90% oxygen treatment of newborn mice is sufficient to produce a chronic deficiency in alveolarization. A means to normalize alveolarization and the significance of, or connection to the temporary septal, tissue area and vascular deficits requires addressing in the future investigations.

## Competing interests

The authors declare they have no competing interests.

## Authors’ contributions

ABF carried out all the experiments and participated in writing the manuscript; TJC helped design the study and participated in the writing of the manuscript; RM designed the study and participated in the writing of the manuscript. All authors read and approved the final manuscript.

## Pre-publication history

The pre-publication history for this paper can be accessed here:

http://www.biomedcentral.com/1471-2466/14/59/prepub
